# Ambipolar Charge Storage in Type‐I Core/Shell Semiconductor Quantum Dots toward Optoelectronic Transistor‐Based Memories

**DOI:** 10.1002/advs.202100513

**Published:** 2021-06-26

**Authors:** Hao hu, Guohao Wen, Jiamin Wen, Long‐Biao Huang, Meng Zhao, Honglei Wu, Zhenhua Sun

**Affiliations:** ^1^ Key Laboratory of Optoelectronic Devices and Systems of Ministry of Education and Guangdong Province College of Physics and Optoelectronic Engineering Shenzhen University Shenzhen 518060 China; ^2^ Jiangsu Key Laboratory of Micro and Nano Heat Fluid Flow Technology and Energy Application School of Physical Science and Technology Suzhou University of Science and Technology Suzhou 215009 China

**Keywords:** InP/ZnS quantum dots, optoelectronic memory, organic transistors, photonic synaptic transistors, type‐I core–shell quantum dots

## Abstract

Efficient charge storage media play a pivotal role in transistor‐based memories and thus are under intense research. In this work, the charge storage ability of type‐I InP/ZnS core/shell quantum dots is well revealed through studying a pentacene‐based organic transistor with the quantum dots (QDs) integrated. The quantum well‐like energy band structure enables the QDs to directly confine either holes or electrons in the core, signifying a dielectric layer‐free nonvolatile memory. Especially, the QDs in this device can be charged by electrons using light illumination as the exclusive method. The electron charging process is ascribed to the photoexcitation process in the InP‐core and the hot holes induced. The QDs layer demonstrates an electron storage density of ≈5.0 × 10^11^ cm^−2^ and a hole storage density of ≈6.4 × 10^11^ cm^−2^. Resultingly, the output device shows a fast response speed to gate voltage (10 µs), large memory window (42 V), good retention (>4.0 × 10^4^ s), and reliable endurance. This work suggests that the core/shell quantum dot as a kind of charge storage medium is of great promise for optoelectronic memories.

## Graphical abstract

Type‐I InP/ZnS core/shell quantum dots are implanted into a pentacene organic transistor. The ambipolar charge storage ability and excellent optoelectronic property of the QDs enable the device to be optically programmed and electrically erased, signify a nonvolatile optoelectronic memory. The device demonstrates good retention, endurance, and fast response speed to the electric bias.

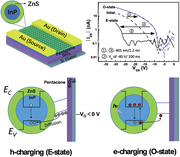

## 1 Introduction

Novel nonvolatile memory devices, such as memristors,^[^ ^1^ ^]^ phase‐change memories,^[^ ^2^ ^]^ ferroelectric memories,^[^ ^3^ ^]^ floating‐gate memories,^[^ ^4^ ^]^ etc., are going through a research upsurge as a response to the eager demand for a solution addressing the “Memory wall” in the modern silicon‐based computing system.^[^ ^5^ ^]^ Thereinto, transistor‐based memories (TBMs) are regarded as an important approach with unique advantages including nondestructive reading, modern integrated circuit architectural compatibility, and reliable switching characteristic.^[^ ^6^ ^]^ TBMs have a relatively straightforward working mechanism, that is, modulation of the conductance of the active layer by the charge storage nearby. In a conventional floating‐gate transistor memory, the charge storage is realized through a bulk conductor (floating‐gate) isolated from the active layer by a dielectric layer. Aiming for a better TBM device, semiconductor nanocrystals have been investigated as effective alternatives to the bulk floating gate, given that their abundant surface states render their charge storage ability huge, and discrete feature ensures a limited charge leakage.^[^ ^7^ ^]^ TBMs using nanocrystals of Si,^[^ ^8^ ^]^ CdSe,^[^ ^9^ ^]^ graphene,^[^ ^10^ ^]^ carbon,^[^ ^11^ ^]^ CsPbBr_3,_^[^ ^12^ ^]^ CdSe/ZnS core/shell,^[^ ^13^ ^]^ etc. as the floating gates demonstrated outstanding figures of merits in terms of speed, endurance, retention, and so on. Meanwhile, thanks to the excellent optoelectronic property of the semiconductor nanocrystals, some output TBMs were endowed with optoelectronic operation ability, that is, the devices could be programmed and/or erased by the light illumination.^[^ ^9^, ^14^ ^]^ Using a light as an operation method of TBMs has advantages including nondestructive operation, multiple data storage, etc.^[^ ^7^, ^15^ ^]^ Moreover, these optoelectronic memories are of particular significance to the emerging all‐optical circuit and sensory neuromorphic computing.^[^ ^14^, ^16^, ^17^, ^18^, ^19^ ^]^

Nevertheless, most reports on optoelectronic TBMs based on semiconductor crystals are using the conventional floating‐gate structure. Charges must travel across a dielectric layer to exchange between the charge storage medium and the active layer, which would hinder the speed, efficiency, and endurance of the device.^[^ ^20^, ^21^ ^]^ In this regard, optoelectronic TBMs without the dielectric layer emerged. In relevant research, one well‐known strategy for charge storage is to take advantage of trap states in either the active layer or the charge storage media.^[^ ^19^, ^22^, ^23^ ^]^ Alternatively, structure‐engineering of colloidal nanomaterials, namely, capping individual nano‐charge storage media with charge blocking layer, have been proved to be another effective way for charge storage.^[^ ^7^, ^9^, ^24^, ^25^ ^]^ To illustrate, Jeong et al. reported that CdSe quantum dots (QDs) capped with selected surface ligands could be applied as effective charge storage media in a dielectric layer free optoelectronic TBM.^[^ ^9^ ^]^ The device demonstrated a fast photo‐induced erasing process due to the efficient charge exchange between the CdSe QDs and the active layer. Previously we have shown that colloidal type‐I core/shell QDs could robustly store charges using its quantum well‐like energy band structure. The charge injection/expelling would happen under a voltage bias. Resultingly, a dielectric layer free TBM with electrical operationality was fabricated.^[^ ^25^ ^]^ Nevertheless, the application potential of the core/shell QDs in TBMs is yet to be exhausted since the optoelectronic property of the material was not referred to. In this study, InP/ZnS core/shell QDs are used to address this issue. The QDs are put atop the active layer of a bottom‐gate‐configuration organic transistor based on pentacene. The type‐I band structure of the core/shell QDs enables them to store both holes and electrons. The p‐type pentacene is nailed down to be the hole supplier, which would inject the holes to the QDs through a diffusion process under a negative gate voltage. On the other hand, light illumination is found to generate electrons in the QD and thus be the only valid method to charge the QDs with electrons in this device. As a result, the device behaves as a good optoelectronic TBM that can be optically programmed and electrically erased. The state of the device can be well controlled by the electrical pulse power and the incident light dose, signifying an application potential of photonic artificial synapses.

## 2 Results and Discussions

**Figure 1:**
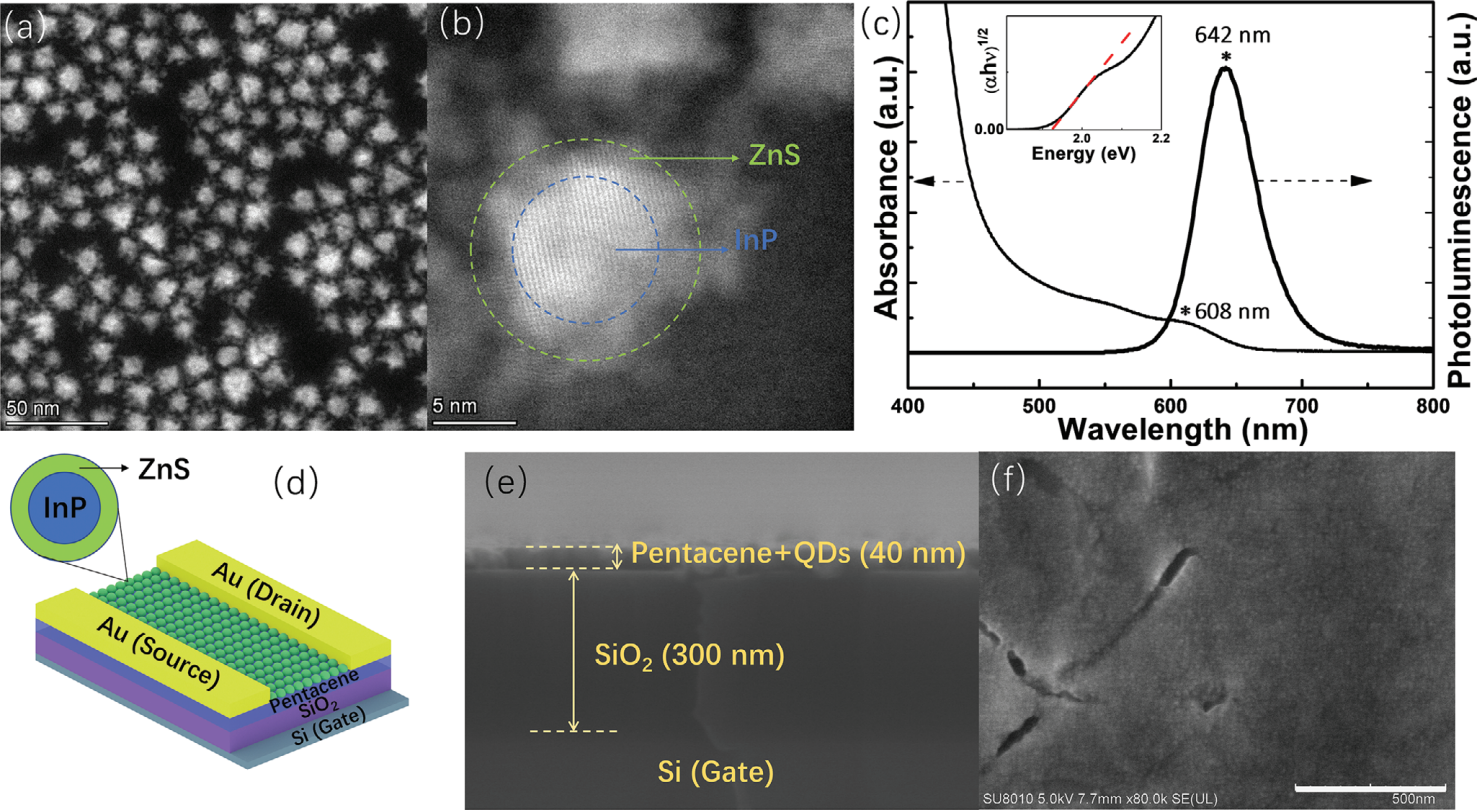
a,b) HAADF‐STEM images of InP/ZnS core–shell QDs. The scale bars in (a) and (b) are 50 nm and 5 nm, respectively; c) Photoluminescence and absorbance spectra of InP/ZnS core–shell QDs in solution. The inset is the Tauc plot based on the absorbance spectrum; d) Structure schematic of the organic transistor‐based memory using pentacene‐QDs bilayer as the active layer; e) Cross‐section SEM image of the channel of the transistor; f) SEM image of the surface of the pentacene‐QDs bilayer film. The scale bar is 500 nm.

The InP/ZnS core/shell QDs were synthesized using a reported method.^[^ ^26^ ^]^ An InP‐core was first synthesized and then wrapped by the ZnS shell through a layer‐by‐layer growth process of Zn and S atoms. The high‐angle annular dark‐field imaging scanning transmission electron microscope (HAADF‐STEM) images of the QDs were acquired and shown in **Figure** **1**a,b. The figures demonstrate obvious contrast between the core and shell, denoting an InP‐core diameter of about 8 nm and a ZnS‐shell thickness of about 3 nm (Figure 1b). Notice that the core/shell QD has an irregular shape, implying that the growth of the QDs is subject to the Stranski–Krastanov growth mechanism.^[^ ^27^, ^28^ ^]^ Although an island‐like shell was grown due to the strain energy induced by the lattice mismatch between the core and shell materials, there is a continuous shell film formed in advance, guaranteeing the complete core/shell structure. The photoluminescence (PL) spectrum of the InP/ZnS QDs in Figure 1c shows an emission peak at 642 nm, further verifying the successful capping of the ZnS shell on the InP‐core, since the bare InP QDs are not luminescent.^[^ ^26^, ^29^ ^]^ The absorbance spectrum of the QDs in Figure 1c demonstrates a first excitonic peak at 608 nm, which corresponds to a bandgap of about 1.9 eV, as revealed by the Tauc plot in the inset of Figure 1c. A TBM was fabricated using a bottom‐gate‐top‐contact structure, as schematically shown in Figure 1d. The active layer of the device was prepared through spin‐coating the InP/ZnS QDs atop a pentacene film with a thickness of about 20 nm, forming a QD‐pentacene bilayer film. The cross‐section scanning electron microscope (SEM) image of the device was acquired and shown in Figure 1e, manifesting a total thickness of about 40 nm of the active layer. Considering the diameter of about 14 nm of the InP/ZnS QDs, it implies a quasi‐single layer close‐packed QDs on top of the pentacene. The SEM image of the bilayer film in Figure 1f confirms the uniform distribution of the QDs on the pentacene, while the pure pentacene film has a smooth surface as shown in Figure S1a, Supporting Information. Moreover, cracks were found in the bilayer film after the deposition of the InP/ZnS QDs, which are ascribed to the damages induced by the spin‐coating process of the toluene solution of QDs. The damages include the dissolution of pentacene induced by the toluene and the impediment to the pentacene crystallization during the post annealing process induced by the implanted QDs.

**Figure 2:**
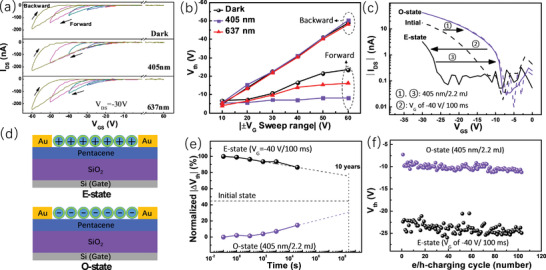
a) Cyclic transfer characteristics of the TBM. The transfer curves were measured with the denoted illumination conditions and *V*_G_ sweep direction. The irradiances of the 405 and 637 nm lights were 3.7 and 9.1 mW cm^−2^, respectively. *V*_DS_ was set to be −30 V for all measurements; b) The curves of *V*_th_ of the transfer curves in (a) versus the *V*_G_ sweep ranges; c) Unidirectional forward transfer curves after bias or illumination operation were applied to the device. Operations ①, ②, and ③ were conducted successively. *V*_DS_ = −30 V; d) Schematics of the charge storage situations in the O‐state and E‐state in (c); e) Normalized *V*_th_ of the E‐state and O‐state transfer curves along with time. The freshly attained E‐state is deemed as 100% and O‐state as 0; f) *V*_th_ monitored while switching the TBM between the O‐state and E‐state more than 100 times.

The transfer characteristics of the TBM device based on the QD‐pentacene bilayer was measured in different regards with a *V*_DS_ of −30 V. **Figure** **2**a shows the cyclic transfer curves of the device with different *V*_G_ sweep ranges. From top to bottom, the three characteristics were separately acquired with the device in the dark, under light illumination of 405 and 637 nm. The irradiances of the 405 and 637 nm lights were 3.7 and 9.1 mW cm^−2^, respectively, which were fixed as the light sources in all the future studies. For each characteristic, the measurement sequence of different curves was from the small sweep range to the large. For all curves, the *V*_G_ swept from positive to negative (forward sweep) and then back to positive (backward sweep) with identical sweep rates. These transfer characteristics all show considerable hysteresis, implying a charge storage effect during the *V*_G_ sweeping. For comparison, control devices with only pentacene or QD film (QD‐only device) as the active layer were also fabricated. For the device with only pentacene, the pentacene was either pristine, or treat with toluene by spin‐coating toluene atop, producing a pristine pentacene device or pentacene‐toluene device, respectively. The structures of the control devices are shown in Figure S1b, Supporting Information. The transfer characteristics of the pristine pentacene and pentacene‐toluene devices with the identical measurement protocol in Figure 2a were obtained and shown in Figure S1c,d, Supporting Information, respectively. The curves in Figure S1c, Supporting Information, all show less hysteresis, indicating neglectable charge storage in the pristine pentacene film under all illumination conditions. The larger current in Figure S1c, Supporting Information, than Figure 2a is accordant with the above‐mentioned film damage induced by the QDs deposition. Likewise, the pentacene‐toluene device shows negligible hysteresis in all transfer characteristics. Notice that the channel current of the pentacene‐toluene device is smaller than the pristine pentacene device but larger than the QD‐pentacene device. It corroborates that toluene‐induced dissolution is a partial reason for the pentacene film damage after QD deposition. The feature of the pentacene‐toluene device suggests that the toluene treatment and the film damage can be excluded from the causes for the charge storage ability of the QD‐pentacene device. Additionally, the transfer curve of the QD‐only device in Figure S1e, Supporting Information, reveals that the QD film is nonconducting. Therefore, it can be concluded that in the QD‐pentacene device, the pentacene film is in charge of charge conducting, and the QD film plays the role of charge storage medium. And the latter is responsible for the hysteresis of the transfer curves. The clockwise hysteresis of the transfer curve is typical in TBM, which is the sign of holes storage under negative *V*_G_ and electrons storage under positive *V*_G_.^[^ ^20^, ^30^ ^]^ Nevertheless, there are some notable features in Figure 2a. For all three conditions, the backward curves leftwards shift along with the increase of the *V*_G_ sweep range, which is indicative of the increase of the stored holes.^[^ ^25^, ^31^ ^]^ In the dark, the forward curves shift leftwards with the increase of the *V*_G_ sweep range as well, which is opposite to the knowledge that the positive *V*_G_ would neutralize the stored holes or induce the electron storage.^[^ ^25^ ^]^ Moreover, each forward curve in the dark is almost at the same position as the previous backward curve, and the misalignment between each forward curve and its previous backward curve increases with the increase of the *V*_G_ sweep range. It suggests that the positive *V*_G_ is seriously incompetent to neutralize the stored holes induced by the negative *V*_G_ with the same intensity,^[^ ^12^ ^]^ while the incompetence keeps being alleviated with the increase of the stored holes. As a comparison, the transfer characteristics under light illumination show that this incompetence could be largely relieved by the illumination of 637 nm and eliminated by 405 nm. Namely, each forward curve under the 637 nm condition is in the right of the last backward curve, and all the forward curves under the 405 nm condition are in the same initial position. The threshold voltages (*V*_th_) of the transfer curves in Figure 2a were extracted and plotted along with the *V*_G_ sweep ranges in Figure 2b to exhibit the position change of the transfer curves. The results show clearly that the neutralization of the holes stored in QDs is efficient under the illumination of 405 nm, less efficient under 637 nm, and least in the dark. Under the 405 nm illumination, the cyclic transfer curve within *V*_G_ of ±60 V produces the largest memory windows of about 42 V.

The memory windows signify the potential of the device to be applied as a TBM. The impacts of the electric bias and light illumination on the device were ascertained by studying the unidirectional forward transfer curves. The initial transfer curve with a *V*_th_ of −13.1 V was first acquired and shown as the dashed line in Figure 2c. Then the light of 405 nm with the irradiance of 3.7 mW cm^−2^ was used to illuminate the device for 150 s, and thus cast a total 2.2 mJ power (405 nm/2.2 mJ) to the channel area. The resulting transfer curve was found rightwards shifted to the position with a *V*_th_ of −6.1 V. The optically induced state of the device is marked as the O‐state from now on. After that, a *V*_G_ pulse with an amplitude of −40 V and width of 100 ms (−40 V/100 ms) was applied to the device. Consequently, the transfer curve shifted to the left of the initial state, with a *V*_th_ of −22.0 V. The electrically induced state is hereinafter marked as the E‐state for short. Again, light illumination (405 nm/2.2 mJ) was applied to the device in the E‐state. As a result, the transfer curve went back to the previous O‐state. The initial transfer curve reflects a state that no charge is stored in the QDs. The rightwards shifted transfer curves, that is, the O‐state, corresponds to the electron storage in the QDs.^[^ ^6^, ^25^, ^30^ ^]^ Oppositely, the E‐state points to the hole storage in the QDs. The charge storage situations in the O‐state and E‐state are illustrated in Figure 2d. The density of the stored charge (*n*) could be estimated through the equation: $n\;=\;\Delta V_{\mathrm{th}}C_{\mathrm{Si}O_{2}}/e$, where Δ*V*_th_ is the *V*_th_ change from the initial state, *e* is the element charge (1.602 × 10^−19^ C), and $C_{\mathrm{Si}O_{2}}$ is the capacitance per unit area of the SiO_2_ dielectric layer. The hole density (*n*_h_) in the E‐state and electron density (*n*_e_) in the O‐state were calculated to be 6.4 × 10^11^ cm^−2^ and 5.0 × 10^11^ cm^−2^, respectively. Furthermore, the QD density was evaluated to be about 3.9 × 10^11^ cm^−2^ through an SEM image shown in Figure S1f, Supporting Information. The charges stored in each QD are thus calculated to be about 1.6 for hole and 1.3 for electron. Hence, there are both singly and multiply charged QDs in the O and E‐states, implying the possibility to further charge the QDs by increasing the bias voltage intensity or light irradiance. It is worthy of noting that the light illumination of 405 nm/2.2 mJ shifted both the initial and E‐state transfer curves to the same O‐state. It implies an upper limit of the electrons that can be stored in the QDs (5.0 × 10^11^ cm^−2^) under the current light irradiance of 3.7 mW cm^−2^, and 405 nm/2.2 mJ is far more than the dose needed to reach the limit from the initial state. During the above measurement, the initial state was hard to fix since any bias or light illumination would induce the change of the device state. Therefore, the O‐state with saturated electron storage was used as the benchmark of the device state. From the O‐state, a *V*_G_ of −40 V/100 ms would neutralize the stored electrons and then induced hole storage in the QDs. The nonvolatility of the charge storage in the QDs was characterized by monitoring the *V*_th_ of the transfer curves in the E‐state and O‐state along with time, with the freshly attained E‐state deemed as 100% and O‐state as 0. The result in Figure 2f shows that the *V*_th_ of the E‐state and O‐state converge to the initial state, indicating the release process of the stored charges. This release is slow, with over 60% of *V*_th_ discrepancy retained after 4 × 10^4^ s. Extrapolating to 10 years, there is still over 30% charges residual in the QDs. The retention performance could be improved by increasing the shell thickness and deliberately choosing the surface ligand of the QDs.^[^ ^9^, ^25^, ^32^ ^]^ Then the endurance of the QDs to the charging process was tested by repeating the hole charging (h‐charging)/electron charging (e‐charging) cycle 100 times. The h‐charging was realized using a *V*_G_ pulse of −40 V/100 ms, and the e‐charging using a light illumination of 405 nm/2.2 mJ. The *V*_th_ of the E‐state and O‐state transfer curves were extracted and shown in Figure 2e, demonstrating a stable endurance of the QDs in this test.

**Figure 3:**
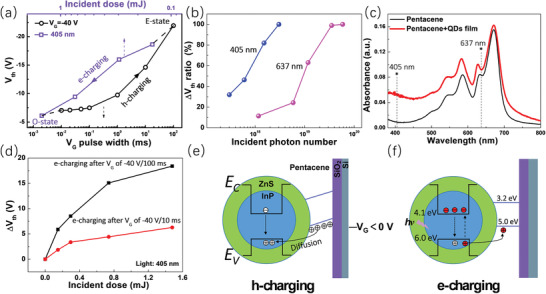
a) Dependence of the *V*_th_ on the width of the *V*_G_ of −40 V in an h‐charging process from the O‐state and on the incident dose of light illumination of 405 nm in the subsequent e‐charging process from the E‐state; b) Δ*V*_th_ ratio in the e‐charging process from the E‐state along with the incident photon numbers for different light sources. The Δ*V*_th_ between the O‐state and E‐state is regarded as 1; c) Absorbance spectra of the active layer of the device before and after the deposition of the QDs; d) Curves of Δ*V*_th_ versus incident light dose of 405 nm in e‐charging processes that started from two different device states. One state is that a *V*_G_ of −40 V/100 ms was applied to the device at the O‐state; the other is that a *V*_G_ of −40 V/10 ms was applied to the device at the O‐state; e) Schematic of the h‐charging process under a negative *V*_G_; f) Schematic of the e‐charging process under an illumination. The red carriers are the photo‐induced hot carriers.

To ascertain the e‐charging process clearly, different incident light doses of 405 nm were applied to the E‐state device in Figure 2c. The induced transfer curves were acquired and shown in Figure S2a, Supporting Information. As expected, the number of the electrons charged into the QDs depends on the incident light dose, and from the E‐state, a light power of 1.5 mJ is sufficient to charge the QDs with electrons of the upper limit number under the current light irradiance. Reversely, the h‐charging process can be precisely controlled by the amplitude or width of the *V*_G_ pulse as well. Figure S2b,c, Supporting Information, show the leftward shifts of the transfer curves from the O‐state upon *V*_G_ pulses of −40 V with variable width and *V*_G_ pulses of 100 ms with variable amplitude, respectively. **Figure** **3**a summarizes the dependence of the *V*_th_ on the width of the *V*_G_ of −40 V in an h‐charging process from the O‐state and the dependence of the *V*_th_ on the incident dose of light illumination of 405 nm in the subsequent e‐charging process from the E‐state. A distinguishable Δ*V*_th_ was attained by a *V*_G_ pulse with a narrow width of 10 µs, manifesting the application potential of the core–shell QDs in fast speed memories.^[^ ^20^, ^21^ ^]^ It is worthy of pointing out that while −40 V/100 ms was used as the strongest h‐charging operation, further increase of the *V*_G_ amplitude or width would induce a larger leftward shift of the transfer curves, indicating for a huge hole accommodation ability of the QDs. Nevertheless, a further leftward shift of the transfer curve beyond the E‐state of −40 V/100 ms made *V*_th_ difficult to determine in the *V*_G_ sweep range of ±30 V, yet a wider *V*_G_ sweep range would disorder the transfer characteristic by bringing out an unexpected h‐charging effect. Therefore, −40 V/100 ms was chosen as the most intensive h‐charging operation in the study on transfer characteristics. Compared with the straightforward electrically induced h‐charging process,^[^ ^9^, ^13^, ^33^ ^]^ the optically induced e‐charging process needs to be addressed better since both the pentacene and QDs are light sensitive. To do so, light illuminations of 637 nm with different incident doses were used to charge the QDs with electrons, with the resulting transfer curves recorded and shown in Figure S2d, Supporting Information. As can be seen, a much larger light dose of 10.9 mJ of 637 nm than the 1.5 mJ of 405 nm was needed to saturate the QDs with electrons from the E‐state. On the basis of Figure S2a,d, Supporting Information, the normalized Δ*V*_th_ were plotted along with the incident photon numbers in Figure 3b, with the Δ*V*_th_ between the O‐state and E‐state regarded as 1. The 405 nm light shows a much larger efficiency than 637 nm to charge the QDs with electrons. To explore the reason, the absorbance spectra of the active layer of the device before and after the deposition of the QDs are shown in Figure 3c, with the 405 and 637 nm located. It suggests that the absorption of the QD‐pentacene film at 405 nm comes from the QD component mostly, and the absorption at 637 nm mainly comes from the pentacene component. Therefore, we infer that the photoexcitation in QDs makes the main contribution to the e‐charging process. Considering the ZnS shell have a bandgap larger than 3.9 eV (bulk bandgap),^[^ ^34^ ^]^ the photoexcitation shall only happen in the InP‐core. Moreover, the e‐charging processes started from different device states were compared to clarify the driving force for the charging. Two different h‐charging operations, that is, *V*_G_ of −40 V/100 ms or −40 V/10 ms, were separately conducted to a device in the O‐state. Then, 405 nm light was used to charge electrons into the device in these two different states. The induced Δ*V*_th_ were plotted along with the incident light dose in Figure 3d, demonstrating a smaller Δ*V*_th_ for the less h‐charged state (−40 V/10 ms) at the same light dose. It means that the e‐charging speed is dependent on the charge density in the QDs.

Given the above, the scenario of the working process of the device was conceived and shown in Figure 3e,f. The InP/ZnS core/shell QDs have been widely revealed to have a quantum well‐like type‐I band structure.^[^ ^26^, ^34^, ^35^, ^36^ ^]^ The valence band and conduction band of the InP‐core are estimated to be about 6.0 and 4.1 eV, respectively.^[^ ^37^ ^]^ The pentacene has a valence band of 5.0 eV and a conduction band of 3.2 eV.^[^ ^14^ ^]^ The thin ZnS shell would have a very large bandgap due to the quantum confinement effect, with a conduction band above the pentacene and a valence band lower than InP‐core.^[^ ^36^ ^]^ A negative *V*_G_ leads to the accumulation of holes in the pentacene due to the capacitive coupling effect. The holes can overcome the energy barrier formed by the ZnS shell as well as the unfavorable valence band alignment between the pentacene and InP‐core and get injected into the InP‐core (Figure 3e). Since the QDs layer is placed atop the pentacene, no bias is supposed to be applied to the QDs during the bottom V_G_ proceeding. Hence, the injection would be motivated only by the diffusion force formed due to the high concentration discrepancy between the QDs and pentacene. If there are electrons stored in the QDs in advance, they will be neutralized first. The ZnS shell with a lower valence band can confine the holes in the InP‐core.^[^ ^25^ ^]^ On the other hand, when a light (405 or 637 nm) illuminates the device, photo‐induced hot electrons and holes are generated in the InP‐core. The holes would transfer to the pentacene, inducing an e‐charging process in the QDs. The previously stored holes (if any) in the QDs would help accelerate the e‐charging process, thanks to the increased hole concentration. As a contrast, Figure S3a,b, Supporting Information, show that a positive *V*_G_ of 40 V cannot induce any change of the transfer curve from its initial state and O‐state, respectively. The reason can be attributed to that the p‐type pentacene can only play the role of a hole supplier,^[^ ^9^, ^31^, ^33^ ^]^ and a positive *V*_G_ would deplete the holes in the pentacene film. Figure S3c, Supporting Information, demonstrates that the transfer curve in the E‐state cannot be changed by a positive *V*_G_ neither, meaning that a positive V_G_ is incompetent to neutralize the stored holes in the QDs. This is accordant with the above analysis of Figure 2a. In principle, the depletion of holes in the pentacene under a positive *V*_G_ would increase the hole concentration discrepancies between the QDs and pentacene. It seems that the diffusion force is not large enough to overcome the barrier between the InP‐core and pentacene even though there is a favorable valence band alignment. Therefore, the diffusion force, the energy of the hot holes, and the favorable valence band alignment may all contribute to the e‐charging process under illumination. Their exact roles need to be addressed further.

**Figure 4:**
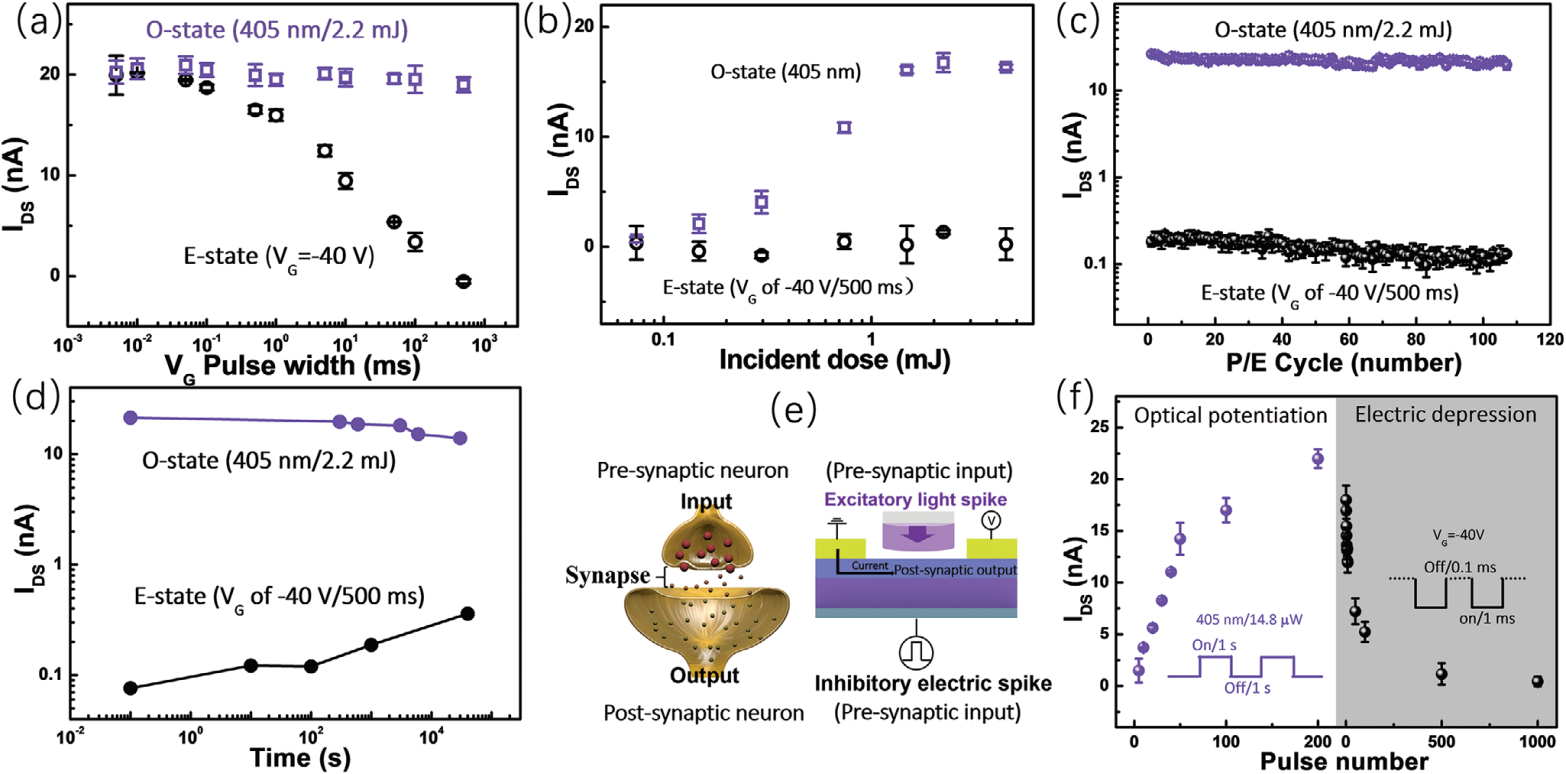
a) Relationship between the *I*_DS_ after h‐charging and the width of the *V*_G_ pulse. *V*_G_ pulses of −40 V with different widths and illumination of 405 nm/2.2 mJ were alternatively applied to the device. *I*_DS_ was recorded after each operation; b) Relationship between the *I*_DS_ after e‐charging and the incident dose of the 405 nm light. Illumination of 405 nm with different incident dose and *V*_G_ pulses of −40 V/500 ms were alternatively applied to the device. *I*_DS_ was recorded after each operation; c) Endurance characteristics of the device. The h‐charging (*V*_G_ of −40 V/500 ms) and e‐charging (405 nm/2.2 mJ) operations were cyclically applied to the device, with the cycles repeated 100 times. *I*_DS_ was recorded after each operation; d) Decays of the *I*_DS_ along with time after the h‐charging (*V*_G_ of −40 V/500 ms) and e‐charging (405 nm/2.2 mJ) operation were separately applied to the device; e) Correspondence between an actual synapse (left) and an artificial synapse (right) based on our device; f) Photonic potentiation‐electric depression cycle of the artificial synapse realized by illumination pulse and negative *V*_G_ pulse.

The channel currents (*I*_DS_) of the transistor upon different charging processes were characterized to explore the performance of the device as a TBM. In all coming study, the I_DS_ were read at *V*_G_ of −20 V and *V*_DS_ of 10 V. To eliminate the impact of the reading process on device state, the *I*_DS_ reading was carried out at a fast sampling speed with only two sampling points. First, h‐charging from the benchmark O‐state was conducted to the device using *V*_G_ pulses of −40V with different widths. After each h‐charging operation, the device was restored to the O‐state using illumination of 405 nm/2.2 mJ. The *I*_DS_ of the device after each h‐charging and restoring were recorded and plotted along with the *V*_G_ pulse width, as shown in **Figure** **4**a. It demonstrates that a distinguishable I_DS_ change can be made by a *V*_G_ of −40 V/50 µs. The writing time at the sub‐millisecond level is consistent with the 10 µs obtained from the above *V*_th_ characteristic (Figure 3a). In the same way, the dependence of the *I*_DS_ after an e‐charging operation on the incident light dose was acquired and shown in Figure 4b. After each e‐charging operation, the *I*_DS_ was restored to an off state using a *V*_G_ of −40 V/500 ms, which was proved necessary to attain an off *I*_DS_ in Figure 4a. As can be seen, the *I*_DS_ becomes stable after 1.5 mJ light was cast to the device, and further illumination would not change the value, which corroborates the above discussion (Figure S2a, Supporting Information). The endurance and retention characteristics of the device were studied by conducting the most intensive h‐charging (*V*_G_ of −40 V/500 ms) and e‐charging (405 nm/2.2 mJ) operations. Figure 4c shows the *I*_DS_ recorded during 100 times successive operation cycles, suggesting a good endurance of the device. The decays of the *I*_DS_ after h‐charging and e‐charging operation were separately recorded along with time and shown in Figure 4d. The *I*_DS_ in two different states demonstrate a limited decay in 3 × 10^4^ s, denoting the robust charge storage in the core/shell QDs. Some reported transistor‐based memories using core/shell QDs as the charge storage media are summarized and compared with the presented one in **Table** **1**. The presented device possesses a simpler structure without the tunneling dielectric layer, benefit from which it can be programmed by a narrower electrical pulse. Meanwhile, the presented device also has a benign information retention performance and optical operationality. The potential of the TBMs in the construction of an artificial neuromorphic system has been widely revealed by using the devices to simulate the main synaptic functions.^[^ ^14^, ^38^, ^39^, ^40^, ^41^, ^42^, ^43^, ^44^ ^]^ The optoelectronic TBMs, which can response to both electric and optical stimuli, possess two approaches to mimic the plasticity of a synapse.^[^ ^11^, ^14^, ^41^, ^43^ ^]^ Figure 4e illustrates the correspondence between an actual synapse (left) and an artificial synapse (right) based on our device. In this regard, light spikes of 405 nm with an intensity of 14.8 µW and duration of 1 s were cast to the channel of the device with a frequency of 0.5 Hz, realizing a photonic potentiation of the artificial synapse. Then, electric spikes of *V*_G_ with an amplitude of −40 V and width of 1 ms were applied to the device with an interval of 0.1 ms to conduct an electric depression. The result in Figure 4f shows that 200 excitatory light spikes and 1000 inhibitory electric spikes manage to achieve a potentiation‐depression cycle in the device.

**Table 1:** Summary of concerned device features of some transistor‐based memories using core/shell QDs as the charge storage media

Core/shell charge storage medium	Tunneling dielectric	Minimum electrical pulse width used [s]	Measured retention [s]	Optical operationality	Ref.
CdSe/ZnSe	None	1.0	8 × 10^3^	Yes	[45]
CdSe/ZnS	PMMA	0.5	5 × 10^3^	No	[46]
Au/SiO_2_	SiO_2_	1.0	1 × 10^5^	No	[24]
CdSe/ZnS	Al_2_O_3_	1.0	1 × 10^4^	Yes	[13]
CdSe/ZnS	Al_2_O_3_	10.0 m	1 × 10^4^	No	[47]
CdSe/ZnS	Al_2_O_3_	10.0 m	1 × 10^4^	No	[48]
Au/Pd	Al_2_O_3_	1.0 m	1 × 10^5^	No	[49]
Au/PS	PS	2.0 m	1 × 10^4^	No	[50]
CdSe/ZnS	Al_2_O_3_	10.0 m	1 × 10^4^	Yes	[51]
ZnSe/ZnS	None	0.1	4.1 × 10^4^	No	[25]
InP/ZnS	None	10.0 µ	4 × 10^4^	Yes	This work

## 3 Conclusions

InP/ZnS core/shell QDs were implanted into an organic transistor based on pentacene, forming direct contact with the active layer. A battery of studies reveals the nonvolatile ambipolar charge storage ability of the QDs, which is ascribed to the type‐I energy band structure that can confine both electrons and holes in the core. A negative gate voltage would accumulate holes in the semiconductor layer and induce hole diffusion to the QDs. The direct contact between the QDs and pentacene leads to a fast electron charging speed, which could establish a distinguishable *V*_th_ change upon a *V*_G_ pulse of −40 V with 10 µs width. On the other hand, the photoexcitation effect in the InP‐core was proved to be exclusively responsible for the electron charging to the QDs. The electrically induced hole charging and optically induced electron charging properties of the QDs enable the device to be applied as a nonvolatile optoelectronic memory. The memory can be programmed by light and erased by negative *V*_G_, demonstrating a memory window of up to 42 V, good retention in 4 × 10^4^ s, and benign endurance. Based on this device, an artificial synapse with optical potentiation and electric depression functions was demonstrated. This work manifests the excellent advantages of the type‐I core/shell QDs as charge storage media in nonvolatile optoelectronic memories.

## 4 Experimental Section

### Materials Preparation

All materials and reagents for QD synthesis were purchased from Sigma‐Aldrich and used as received without further purification. Pentacene for thermal evaporation was purchased from Xi'an Baolaite Ltd. InP/ZnS core/shell QDs solution in toluene were synthesized using a reported method.^[^ ^26^ ^]^ Indium (III) chloride (100 mg, 0.45 mmol) and zinc (II) chloride (300 mg, 2.2 mmol) were mixed in oleylamine (5.0 mL). The mixture was degassed at 120 °C for an hour under stirring. The solution was then heated to 180 °C under an Argon atmosphere. Tris‐(diethylamino)phosphine (0.45 mL, 1.6 mmol) was quickly injected into the solution swiftly, so began the growth process of the InP nano‐core. On the other hand, sulfur (S) powder was solved in Trioctylphosphine (TOP) to get the S precursor with a concentration of about 2.2 mol L^−1^ in advance. Zinc (Zn) precursor was obtained by dissolving Zn(stearate)_2_ in octadecene (ODE), forming a solution of 250mg mL^−1^. At the 20th min of the growth process of the InP‐core, 1 mL S precursor was injected into the solution. Since the 60th min, the temperature was increased to 200 °C. At the 120th min, 4 mL Zn precursor was injected into the solution. Then the temperature was increased to 220 °C. At the 150th min, 0.7 mL S precursor was injected, then the temperature was increased to 240 °C. At the 180th min, 2 mL Zn precursor was injected, then the temperature was increased to 260 °C. At the 210th min, the temperature was cooled down to room temperature. The synthesized InP/ZnS QDs were then precipitated by adding ethanol and the following centrifugation. The supernatant was discarded. The precipitation was dissolved in toluene. This process was repeated once. The final solution was ready to use then.

### Device Fabrications

Si/SiO_2_ wafer with heavily p‐doping Si and SiO_2_ of 300 nm thickness was used as the substrate. P‐type pentacene film with a thickness of 20 nm was thermally evaporated on the cleaned substrate as a semiconductor channel. 50 nm Au electrodes were deposited as the source and drain electrodes on the surface of the film by thermal evaporation through a shadow mask defining a length/width of 0.2/2mm. Then in a glovebox filled with nitrogen, 5 mg mL^−1^ InP@ZnS QDs solution was spin‐coated at a speed/duration of 2000 rpm/60 s on the top of the semiconductor layer. The film was then annealed at 60 °C for 30 min.

### Characterizations

High‐angle annular dark‐field imaging scanning transmission electron microscope (HAADF‐STEM) images were acquired on an FEI Titan Cubed Themis G201 double spherical aberration‐corrected transmission electron microscope with an acceleration voltage of 300 kV. Absorbance spectra were measured using a GBC Cintra2020 spectrometer with tunable wavelengths. Photoluminescence (PL) spectra were obtained on Spectrofluorometer FS5 (Edinburgh instruments). Scanning electron microscope (SEM) images were obtained using a HITACHI SU8010 scanning electron microscope. Electrical characteristics of the transistors were measured using the combination of a Keithley 4200SCS parameter analyzer and a probe station. CHI continuous lasers with wavelengths of 405 or 637 nm were used for the relevant experiments. The electrical and optical measurements conducted on the devices were carried out in a glovebox filled with nitrogen.

### Statistical Analysis

19 TBMs with identical channel area of 0.4 mm^2^ were fabricated and characterized for their transfer characteristics in Figure 2a. They demonstrated the same feature with diverse exact values. Thus, a representative device was used to carry out the systematical characterization. The data for Figure 4a,b,c,f were presented as the mean ± standard error using the raw data from one time measurement of the same device. The calculation of the standard error was carried out using the embedded function in the EXCEL (Office 365, Microsoft Corporation, USA). The normalization processing was conducted using the normalization function of the Origin8 software (OriginLab Corporation, Northampton, USA). All Graphical presentations were performed by the Origin8 software.

## Conflict of Interest

The authors declare no conflict of interest.

## Supporting information

Supporting Information

## Data Availability

Research data are not shared.

## References

[1] R. Waser, M. Aono, Nat. Mater. 2007, 6, 833. 17972938 10.1038/nmat2023

[2] H. F. Hamann, M. O'Boyle, Y. C. Martin, M. Rooks, H. K. Wickramasinghe, Nat. Mater. 2006, 5, 383. 16604077 10.1038/nmat1627

[3] A. Q. Jiang, W. P. Geng, P. Lv, J.‐w. Hong, J. Jiang, C. Wang, X. J. Chai, J. W. Lian, Y. Zhang, R. Huang, D. W. Zhang, J. F. Scott, C. S. Hwang, Nat. Mater. 2020, 19, 1188. 32541933 10.1038/s41563-020-0702-z

[4] Z. Y. Su, J. Wu, Y. Yao, M. Z. Lin, Z. Y. Ye, P. F. Wang, in IEEE 12th Int. Conf. on ASIC (ASICON), IEEE, Piscataway, NJ 2017, p. 195.

[5] A. Chen, Solid‐State Electron. 2016, 125, 25.

[6] X.‐J. She, D. Gustafsson, H. Sirringhaus, Adv. Mater. 2017, 29, 1604769. 10.1002/adma.201604769 28004860

[7] Z. Lv, Y. Wang, J. Chen, J. Wang, Y. Zhou, S.‐T. Han, Chem. Rev. 2020, 120, 3941. 32202419 10.1021/acs.chemrev.9b00730

[8] M. Olmedo, C. Wang, K. Ryu, H. Zhou, J. Ren, N. Zhan, C. Zhou, J. Liu, ACS Nano 2011, 5, 7972. 21902187 10.1021/nn202377f

[9] Y. J. Jeong, D.‐J. Yun, S. H. Noh, C. E. Park, J. Jang, ACS Nano 2018, 12, 7701. 30024727 10.1021/acsnano.8b01413

[10] Y. R. Kim, Y. E. Jo, Y. S. Shin, W. T. Kang, Y. H. Sung, U. Y. Won, Y. H. Lee, W. J. Yu, Appl. Phys. Lett. 2015, 106, 103105.

[11] Z. Y. Lv, M. Chen, F. S. Qian, V. A. L. Roy, W. B. Ye, D. H. She, Y. Wang, Z. X. Xu, Y. Zhou, S. T. Han, Adv. Funct. Mater. 2019, 29, 1902374.

[12] W. Jiamin, H. Hao, W. Guohao, W. Shuhan, S. Zhenhua, Y. Shuai, J. Phys. D: Appl. Phys. 2021, 54, 114002.

[13] S.‐T. Han, Y. Zhou, L. Zhou, Y. Yan, L.‐B. Huang, W. Wu, V. A. L. Roy, J. Mater. Chem. C 2015, 3, 3173.

[14] Y. Wang, Z. Y. Lv, J. R. Chen, Z. P. Wang, Y. Zhou, L. Zhou, X. L. Chen, S. T. Han, Adv. Mater. 2018, 30, 1802883. 10.1002/adma.201802883 30063261

[15] T. Leydecker, M. Herder, E. Pavlica, G. Bratina, S. Hecht, E. Orgiu, P. Samorì, Nat. Nanotechnol. 2016, 11, 769. 27323302 10.1038/nnano.2016.87

[16] W. Bogaerts, D. Pérez, J. Capmany, D. A. B. Miller, J. Poon, D. Englund, F. Morichetti, A. Melloni, Nature 2020, 586, 207. 33028997 10.1038/s41586-020-2764-0

[17] C. Rios, M. Stegmaier, P. Hosseini, D. Wang, T. Scherer, C. D. Wright, H. Bhaskaran, W. H. P. Pernice, Nat. Photonics 2015, 9, 725.

[18] K. Roy, A. Jaiswal, P. Panda, Nature 2019, 575, 607. 31776490 10.1038/s41586-019-1677-2

[19] Q. S. Wang, Y. Wen, K. M. Cai, R. Q. Cheng, L. Yin, Y. Zhang, J. Li, Z. X. Wang, F. Wang, F. M. Wang, T. A. Shifa, C. Jiang, H. Yang, J. He, Sci. Adv. 2018, 4, eaap7916. 29770356 10.1126/sciadv.aap7916 PMC5954648

[20] P. F. Wang, X. Lin, L. Liu, Q. Q. Sun, P. Zhou, X. Y. Liu, W. Liu, Y. Gong, D. W. Zhang, Science 2013, 341, 640. 23929978 10.1126/science.1240961

[21] C. S. Liu, X. Yan, X. F. Song, S. J. Ding, D. W. Zhang, P. Zhou, Nat. Nanotechnol. 2018, 13, 404. 29632398 10.1038/s41565-018-0102-6

[22] T. Ahmed, S. Kuriakose, S. Abbas, M. J. S. Spencer, M. A. Rahman, M. Tahir, Y. R. Lu, P. Sonar, V. Bansal, M. Bhaskaran, S. Sriram, S. Walia, Adv. Funct. Mater. 2019, 29, 1901991.

[23] Z. Sun, J. Li, C. Liu, S. Yang, F. Yan, Nano Lett. 2021, 21, 723. 33373246 10.1021/acs.nanolett.0c04370

[24] Y. S. Park, J. S. Lee, Adv. Mater. 2015, 27, 706. 25475911 10.1002/adma.201404625

[25] C. Y. Yan, J. M. Wen, P. Lin, Z. H. Sun, Small 2019, 15, 1804156. 10.1002/smll.201804156 30480357

[26] M. D. Tessier, D. Dupont, K. De Nolf, J. De Roo, Z. Hens, Chem. Mater. 2015, 27, 4893.

[27] B. Ji, Y. E. Panfil, N. Waiskopf, S. Remennik, I. Popov, U. Banin, Nat. Commun. 2019, 10, 2. 30602734 10.1038/s41467-018-07837-z PMC6315019

[28] B. Ji, S. Koley, I. Slobodkin, S. Remennik, U. Banin, Nano Lett. 2020, 20, 2387. 32134676 10.1021/acs.nanolett.9b05020 PMC7467768

[29] K. R. Reid, J. R. McBride, N. J. Freymeyer, L. B. Thal, S. J. Rosenthal, Nano Lett. 2018, 18, 709. 29282985 10.1021/acs.nanolett.7b03703 PMC6163126

[30] S. Wang, C. He, J. Tang, X. Lu, C. Shen, H. Yu, L. Du, J. Li, R. Yang, D. Shi, G. Zhang, Adv. Electron. Mater. 2019, 5, 1800726.

[31] M. D. Yi, M. Xie, Y. Q. Shao, W. Li, H. F. Ling, L. H. Xie, T. Yang, Q. L. Fan, J. L. Zhu, W. Huang, J. Mater. Chem. C 2015, 3, 5220.

[32] A. Marent, M. Geller, A. Schliwa, D. Feise, K. Pötschke, D. Bimberg, N. Akçay, N. Öncan, Appl. Phys. Lett. 2007, 91, 242109.

[33] W. Li, F. N. Guo, H. F. Ling, H. Liu, M. D. Yi, P. Zhang, W. J. Wang, L. H. Xie, W. Huang, Small 2018, 14, 1701437. 10.1002/smll.201701437 29165914

[34] C. d. M. Donegá, Chem. Soc. Rev. 2011, 40, 1512.20972490

[35] A. Brodu, M. V. Ballottin, J. Buhot, D. Dupont, M. Tessier, Z. Hens, F. T. Rabouw, P. C. M. Christianen, C. de Mello Donega, D. Vanmaekelbergh, Phys. Rev. B 2020, 101, 125413.

[36] E. Jang, Y. Kim, Y.‐H. Won, H. Jang, S.‐M. Choi, ACS Energy Lett. 2020, 5, 1316.

[37] H. Fu, A. Zunger, Phys. Rev. B 1997, 56, 1496.

[38] S. Wang, L. Liu, L. Gan, H. Chen, X. Hou, Y. Ding, S. Ma, D. W. Zhang, P. Zhou, Nat. Commun. 2021, 12, 53. 33397907 10.1038/s41467-020-20257-2 PMC7782550

[39] X. Hou, C. S. Liu, Y. Ding, L. Liu, S. Y. Wang, P. Zhou, Adv. Sci. 2020, 7, 2002072. 10.1002/advs.202002072 PMC7610317 33173738

[40] H. F. Ling, N. X. Wang, A. N. Yang, Y. H. Liu, J. J. Song, F. Yan, Adv. Mater. Technol. 2019, 4, 1900471.

[41] J. Sun, S. Oh, Y. Choi, S. Seo, M. J. Oh, M. Lee, W. B. Lee, P. J. Yoo, J. H. Cho, J. H. Park, Adv. Funct. Mater. 2018, 28, 1804397.

[42] Y. Chen, W. J. Qiu, X. W. Wang, W. R. Liu, J. X. Wang, G. Z. Dai, Y. B. Yuan, Y. L. Gao, J. Sun, Nano Energy 2019, 62, 393.

[43] M. Lee, W. Lee, S. Choi, J. W. Jo, J. Kim, S. K. Park, Y. H. Kim, Adv. Mater. 2017, 29, 1700951. 10.1002/adma.201700951 28514064

[44] Y. Yu, Q. H. Ma, H. F. Ling, W. Li, R. L. Ju, L. Y. Bian, N. E. Shi, Y. Qian, M. D. Yi, L. H. Xie, W. Huang, Adv. Funct. Mater. 2019, 29, 1904602.

[45] M.‐Y. Chiu, C.‐C. Chen, J.‐T. Sheu, K.‐H. Wei, Org. Electron. 2009, 10, 769.

[46] Y.‐C. Chen, C.‐Y. Huang, H.‐C. Yu, Y.‐K. Su, J. Appl. Phys. 2012, 112, 034518.

[47] D. Hu, X. Wang, H. Chen, T. Guo, Adv. Funct. Mater. 2017, 27, 1703541.

[48] D. Hu, G. Zhang, H. Yang, J. Zhang, C. Chen, S. Lan, H. Chen, T. Guo, IEEE Trans. Electron Devices 2017, 64, 3816.

[49] Y. Zhou, L. Zhou, Y. Yan, S.‐T. Han, J. Zhuang, Q.‐J. Sun, V. A. L. Roy, J. Mater. Chem. C 2017, 5, 8415.

[50] K. Wang, H. Ling, Y. Bao, M. Yang, Y. Yang, M. Hussain, H. Wang, L. Zhang, L. Xie, M. Yi, W. Huang, X. Xie, J. Zhu, Adv. Mater. 2018, 30, 1800595. 10.1002/adma.201800595 29782682

[51] X. Wu, S. Lan, D. Hu, Q. Chen, E. Li, Y. Yan, H. Chen, T. Guo, J. Mater. Chem. C 2019, 7, 9229.

