# Supplementary material for: Ambipolar Charge Storage in Type‐I Core/Shell Semiconductor Quantum Dots toward Optoelectronic Transistor‐Based Memories
Source: Adv Sci (Weinh). 2021 Jun 26;8(16):2100513. doi: 10.1002/advs.202100513 (PMC8373160; doi:10.1002/advs.202100513)
Supplement: Supplementary file 1 — Supporting Information [file ADVS-8-2100513-s001.pdf]

© 2021 Wiley-VCH GmbH

ADVANCED SCIENCE
Open Access

Supporting Information

for *Adv. Sci.*, DOI: 10.1002/advs.202100513

Ambipolar Charge Storage in Type- ⅠCore/Shell Semiconductor Quantum Dots Towards Optoelectronic Transistor-Based Memories

*Hao hu, Guohao Wen, Jiamin Wen, Long-Biao Huang, Meng Zhao, Honglei Wu and Zhenhua Sun**

Supporting Information

Ambipolar Charge Storage in Type- ⅠCore/Shell Semiconductor Quantum Dots Towards Optoelectronic Transistor-Based Memories

*Hao hu, Guohao Wen, Jiamin Wen, Longbiao Huang, Meng Zhao, Honglei Wu, and Zhenhua Sun**

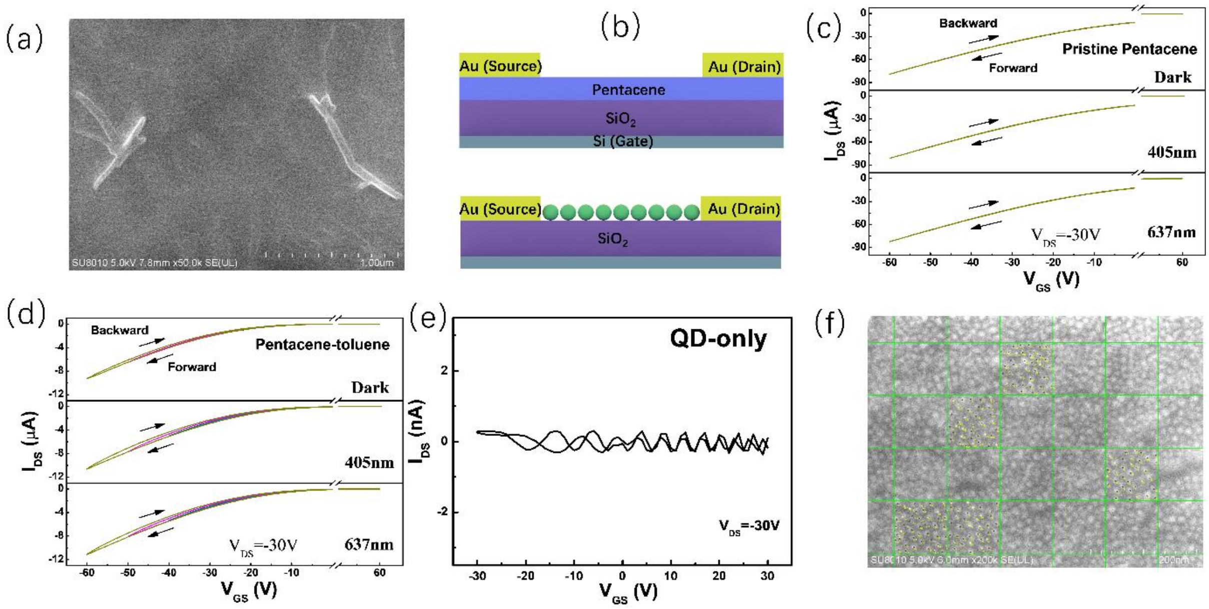

Figure S1. (a) SEM image of the surface of the pristine pentacene film; (b) Structure schematic of the control devices with only pentacene (top) or QD film (bottom) as the active; Transfer characteristics of the pristine pentacene device (c) and pentancene-toluene device (d) with the identical measurement protocol in Figure 2a; (e) Transfer characteristic of the QD-only device; (f) SEM image of the pentacene-QDs film. The image is divided by the green lines, forming 20 complete square grids of 100 nm×100 nm. Five grids are chosen to count the QD density, with each yellow point respresents one QD. The final QD density value is obtained by averaging the results of the five counting.

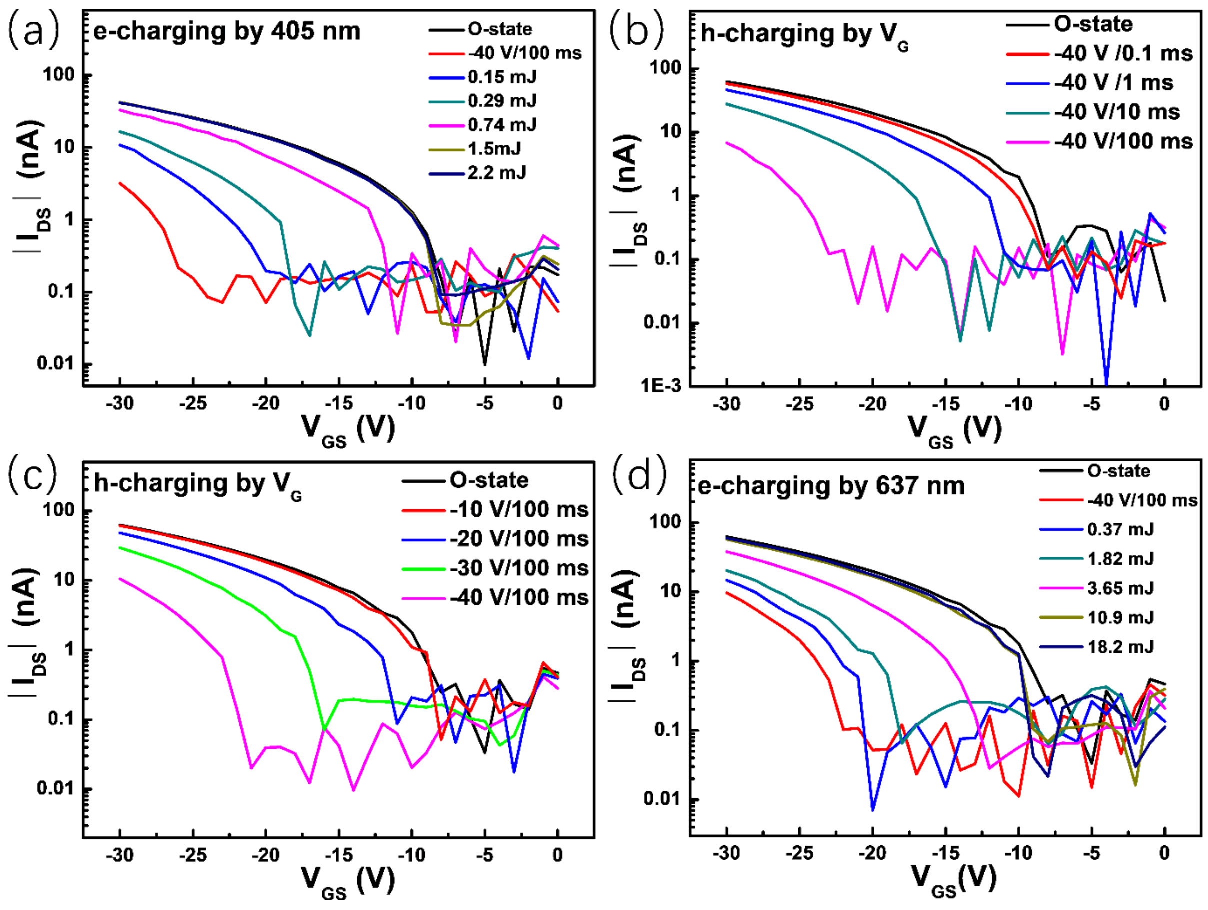

Figure S2. (a) Transfer curves induced by different incident light doses of 405 nm from the E-state.; Transfer curves after V_G pulses of -40 V with variable width (b) and V_G pulses of 100 ms with variable amplitude (c) were applied to the device in the O-state; (d) Transfer curves induced by different incident light doses of 637 nm from the E-state. All the transfer curves in this figure were obtained with a V_{DS} of -30 V.

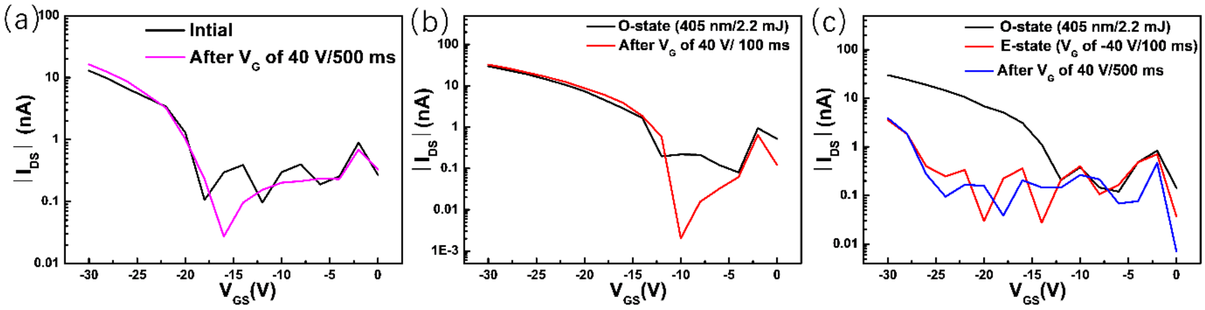

Figure S3. The transfer curves before and after a positive V_G of -40 V applied to the device at initial state (a), O-state (b), and E-state (c). The curves in each figure were acquired in sequence.
